# Multiple Host Barriers Restrict Poliovirus Trafficking in Mice

**DOI:** 10.1371/journal.ppat.1000082

**Published:** 2008-06-06

**Authors:** Sharon K. Kuss, Chris A. Etheredge, Julie K. Pfeiffer

**Affiliations:** Department of Microbiology, University of Texas Southwestern Medical Center, Dallas, Texas, United States of America; University of Freiburg, Germany

## Abstract

RNA viruses such as poliovirus have high mutation rates, and a diverse viral population is likely required for full virulence. We previously identified limitations on poliovirus spread after peripheral injection of mice expressing the human poliovirus receptor (PVR), and we hypothesized that the host interferon response may contribute to the viral bottlenecks. Here, we examined poliovirus population bottlenecks in PVR mice and in PVR mice that lack the interferon α/β receptor (PVR-IFNAR^−/−^), an important component of innate immunity. To monitor population dynamics, we developed a pool of ten marked polioviruses discriminated by a novel hybridization-based assay. Following intramuscular or intraperitoneal injection of the ten-virus pool, a major bottleneck was observed during transit to the brain in PVR mice, but was absent in PVR-IFNAR^−/−^ mice, suggesting that the interferon response was a determinant of the peripheral site-to-brain bottleneck. Since poliovirus infects humans by the fecal–oral route, we tested whether bottlenecks exist after oral inoculation of PVR-IFNAR^−/−^ mice. Despite the lack of a bottleneck following peripheral injection of PVR-IFNAR^−/−^ mice, we identified major bottlenecks in orally inoculated animals, suggesting physical barriers may contribute to the oral bottlenecks. Interestingly, two of the three major bottlenecks we identified were partially overcome by pre-treating mice with dextran sulfate sodium, which damages the colonic epithelium. Overall, we found that viral trafficking from the gut to other body sites, including the CNS, is a very dynamic, stochastic process. We propose that multiple host barriers and the resulting limited poliovirus population diversity may help explain the rare occurrence of viral CNS invasion and paralytic poliomyelitis. These natural host barriers are likely to play a role in limiting the spread of many microbes.

## Author Summary

RNA viruses are highly error prone, and can use their replication infidelity to adapt to complex environments within an infected host. However, viral populations may experience bottlenecks, which limit their diversity and potentially reduce their virulence. We hypothesized that natural barriers may limit the spread of RNA viruses within an infected host. To test this hypothesis, we engineered a pool of ten marked polioviruses identifiable by a novel assay, infected susceptible mice by injection or oral inoculation, and determined the percentage of the ten viruses that successfully spread to various body sites, including the brain. We found that, on average, only 10%–20% of the input viruses were found in most tissues, suggesting that barriers prevented the spread of the whole population. The importance of one such physical barrier, the colonic epithelium, was demonstrated in experiments where the colon was damaged prior to oral inoculation. Under these conditions, 30%–50% of the input viruses successfully spread to various body sites. We propose that host barriers limit viral spread, and this could possibly explain the rare incidence of paralytic poliomyelitis due to central nervous system invasion.

## Introduction

RNA viruses undergo error-prone replication and exist as quasispecies due to the high error rate of RNA-dependent RNA polymerases (RdRp). Within these complex viral populations, genomes can differ by one to many nucleotides resulting from approximately one mutation incorporated per 10,000 nucleotides [1],[2],[3]. For poliovirus, a mutant virus with a high fidelity RdRp attenuated the virus in mice suggesting that a diverse quasispecies is required for full virulence [4],[5]. Genetic recombination also contributes to quasispecies diversity, and has been detected in poliovirus isolated from patients with paralytic poliomyelitis [6]. Mutation and genetic recombination may contribute to greater viral population diversity leading to increased virulence [1],[6],[7].

Poliovirus is an enterovirus spread by fecal-oral transmission and can cause poliomyelitis in humans. Only ∼1% of people infected with poliovirus develop paralytic poliomyelitis from viral invasion of the central nervous system (CNS) [8],[9],[10]. Reversion of the live-attenuated Sabin oral polio vaccine (OPV) by mutation or recombination occurs rather frequently, but only causes vaccine-associated paralytic poliomyelitis (VAPP) in a very small percentage (0.0001%) of people that receive OPV [11],[12],[13],[14]. The reason for such a low incidence of paralytic poliomyelitis and VAPP remains unclear. Interestingly, in human VAPP patients, viral isolates found in the CNS are a minor subset of those found in feces, suggesting viral transit from the gut to the CNS may be difficult in humans [15].

Poliovirus receptor (PVR)-expressing mice are susceptible to poliovirus via intravenous, intraperitoneal, intracerebral, and intramuscular routes [16],[17],[18]. Following intramuscular injection, poliovirus traffics to the CNS by retrograde neuronal transport [19],[20]. Intravenously injected poliovirus is thought to reach the CNS by the blood route, independent of the presence of PVR [21]. Intraperitoneally injected poliovirus may reach the CNS by blood or neural routes. However, these injection models may not mimic the natural fecal-oral route of infection since PVR mice are not orally susceptible. Recently, PVR mice lacking the interferon α/β receptor (PVR-IFNAR^−/−^), a major component of innate immunity, demonstrated oral susceptibility to poliovirus [22],[23]. Oral poliovirus infection in PVR-IFNAR^−/−^ mice resulted in dissemination of virus to many tissues such as esophagus, nasopharynx-associated lymphoid tissue, small intestine, spinal cord, and plasma, as measured by viral titer assay [23]. Viral titers in PVR-IFNAR^−/−^ mice were typically 100 to 10,000-fold higher than titers in PVR mice expressing IFNAR. Here, we use PVR-IFNAR^−/−^ mice to measure bottlenecks faced by the viral population during trafficking inside a host.

Previously, we identified bottlenecks in PVR mice that limited poliovirus population diversity after peripheral injection by intravenous (IV), intraperitoneal (IP), and intramuscular (IM) routes. An artificial quasispecies of four viruses with distinct genomic restriction enzyme site tags were injected, and upon disease onset, brains contained an average of 1.7 input viruses suggesting that an intra-host bottleneck was encountered during trafficking to the CNS [24]. Barriers encountered during spread of microbes are common for many pathogens. Bottlenecks have been described for plant RNA viruses [25], fungi [26], and bacteria such as *Salmonella* and *Yersinia* [27],[28],[29]. Interestingly, the picornavirus foot-and-mouth disease virus, may encounter inter-host and intra-host bottlenecks [30],[31],[32].

In this study, we introduce a new system for monitoring viral quasispecies trafficking in a murine host orally susceptible to poliovirus. We developed a hybridization-based assay for detection of a population consisting of ten marked viruses. To corroborate our previous work, we examined viral trafficking following peripheral injection of PVR mice *vs.* PVR-IFNAR^−/−^ mice. In addition, we orally inoculated PVR-IFNAR^−/−^ mice to follow viral trafficking from the initial inoculation site, the oral cavity, to the gastrointestinal (GI) tract, blood, and brain. We identified several bottlenecks that limit poliovirus spread following oral inoculation, and found means of overcoming some of these barriers by use of a colon-damaging agent.

## Results

### A novel viral population diversity assay

Bottlenecks were previously studied using restriction enzyme site markers in the genomes of four distinct viruses; however, this assay was labor intensive and only included four pool members [24]. To overcome these drawbacks, we developed a more streamlined assay based on signature-tagged mutagenesis technology used in bacterial pathogenesis studies [33]. Hybridization-based detection, 96-well format, and an increased number of pool members are advantages of the new assay.

**Figure 1:**
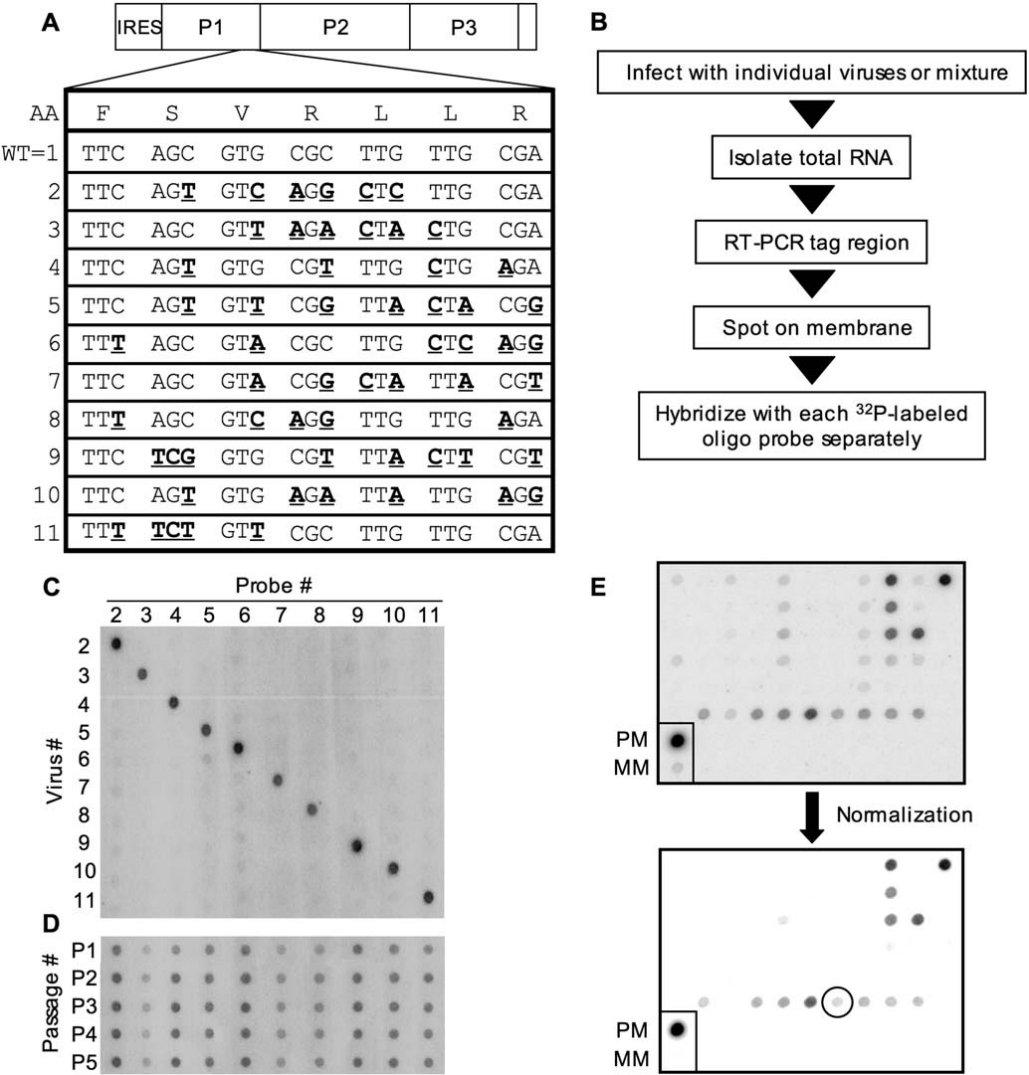
A new hybridization-based quasispecies detection assay. (A) Poliovirus genome highlighting VP3 region of P1 where point mutations were incorporated by site-directed mutagenesis (bold, underlined) to distinguish each of the ten viruses from one another. The amino acid sequence and the wild-type poliovirus genome sequence of the region are shown. Note: Wild type is virus #1, and was not included in our ten-virus pool due to cross hybridization (data not shown). (B) Strategy for the assay. (C) Blot showing the specificity of each labeled probe for its respective viral RT-PCR product. HeLa cells were infected with each individual virus, and samples were processed as described in B. (D) Serial passage competition experiment. HeLa cells were infected with equal amounts of each of the ten viruses, and amplified virus was harvested after a single cycle of replication and used to infect fresh cells. This cycle was repeated for a total of five passages to rule out viral growth defects. (E) Normalization process to eliminate probe cross-reactivity. Signal intensity levels were adjusted such that mismatch (MM; all PCR products except for the one specific for the probe) signal was no longer detectable, and perfect match (PM; PCR product specific for the probe) was used as a positive control. Any visible dots, regardless of intensity, were counted as positive in diversity assays (see circled example).

The artificial quasispecies pool of ten members was engineered by incorporating silent mutations into the VP3 capsid-coding region of the genome, and oligonucleotide probes were designed for specific recognition of each variant (Figure 1A, Figure S1). To determine the specificity of the new assay, HeLa cells were infected with individual viruses or a pool of all ten viruses, RNA was isolated after one replication cycle, and RT-PCR products derived from the RNA were blotted on a nylon membrane using a 96-well vacuum manifold. Oligonucleotide probes were ^32^P-labeled and hybridized to each blotted membrane individually (Figure 1B). Each blot was hybridized with only one labeled probe; therefore, ten blots were performed for each sample. Figure 1C displays the probe hybridization specificity following infection of HeLa cells and probing all samples with each probe. All oligonucleotide probes proved specific for their cognate virus. To ensure the viruses had no detectable growth defects, single-cycle growth curves were performed for each virus and no differences in growth were observed (Figure S2). Additionally, a serial passage competition experiment was performed by infecting HeLa cells with a mixture of the ten viruses and then passaging the virus mixture five times, followed by assessment of input virus loss over time. All ten viruses were maintained throughout the passages, and therefore, no major growth defects of the marked viruses were detected *in vitro* (Figure 1D). For each hybridization assay, normalization was performed to eliminate cross-reactivity of nonspecific probes (Figure 1E). Perfectly matched product (PCR product specific for the probe) and mismatched products (all PCR products except for the one specific for the probe) were loaded on each membrane as controls. The image intensity level of the blots was adjusted until the mismatched product signal became undetectable, revealing only legitimate signals.

### The bottleneck between the periphery and brain following injection is reduced in PVR-IFNAR^−/−^ mice

**Figure 2:**
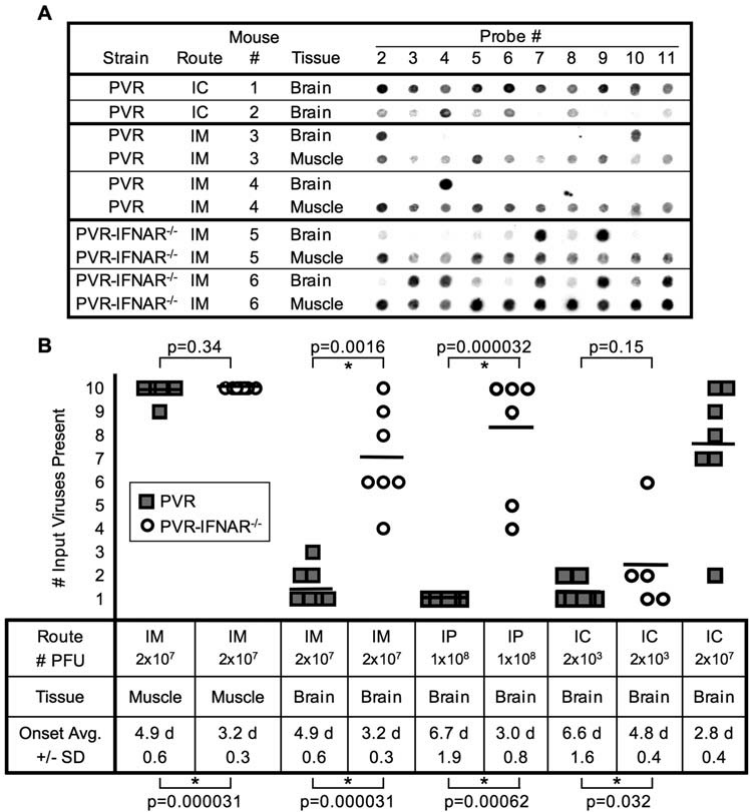
Viral diversity in injected PVR mice and PVR-IFNAR^−/−^ mice. (A) PVR mice were injected intracerebrally (IC) and intramuscularly (IM) with 2×10^7^ PFU of the ten-virus pool, tissues were harvested upon disease onset, and viral diversity in brain and muscle was determined and compared to PVR-IFNAR^−/−^ mice injected IM. Blots derived from two representative viral-derived RT-PCR products are shown per condition, with specific probes numbered along the top. (B) Number of input viruses present in tissues from IM, intraperitoneally (IP), or IC-injected PVR mice and PVR-IFNAR^−/−^ mice. Data from experiments shown as representative examples in panel A and additional experiments, are compiled to display results from at least 5 mice per condition. PVR mice, gray squares; PVR-IFNAR^−/−^ mice, open circles. Horizontal bars denote the average for each group. The average time of symptom onset (day post-inoculation) +/− standard deviation is shown at the bottom. The p values from Student's *t* test are shown: values above the graph compare viral diversity among indicated samples, values below the graph compare disease onset time among indicated samples. An asterisk denotes statistical significance (p<0.05).

Validation of the new hybridization assay confirmed the bottleneck effect observed in previous experiments [24]. PVR mice were inoculated with 2×10^7^ plaque-forming units (PFU) of a pool of all ten viruses (2×10^6^ PFU each; viruses 2 through 11) by intramuscular (IM) or intracerebral (IC) injection. Brains of mice inoculated with 2×10^7^ PFU by the IC route contained most, if not all, input viruses upon disease onset; however, the brains of IM-injected mice contained 10% to 30% of the input viruses (Figure 2A, 2B). For IM-injected mice, all ten viruses were present at the inoculation site, muscle. Brains of PVR mice inoculated by the intraperitoneal (IP) route with 1×10^8^ PFU of the ten-virus pool contained only 10% of the input viruses. These experiments validated the new assay and confirmed our previous results [24].

Next, we measured viral population diversity in PVR-IFNAR^−/−^ mice, which are hyper-susceptible to poliovirus [22],[23]. We hypothesized that innate immunity may contribute to the bottleneck, and therefore, we predicted increased population diversity in the brains of PVR-IFNAR^−/−^ mice. PVR-IFNAR^−/−^ mice were injected intramuscularly with 2×10^7^ PFU of the ten-member pool. As shown in Figure 2A and 2B, the brain bottleneck was greatly diminished in PVR-IFNAR^−/−^ mice, with 40% to 100% of the input viruses detectable in the brain. In fact, the brains of IM-injected PVR-IFNAR^−/−^ mice contain an average of 70% of the input viruses, a result comparable to PVR mice injected IC with 2×10^7^ PFU. Similarly, brains of IP-inoculated PVR-IFNAR^−/−^ mice contained 80% of the input viruses. The diminished bottleneck in PVR-IFNAR^−/−^ mice may be the result of increased peripheral titers in PVR-IFNAR^−/−^ mice, essentially increasing the viral dose, physical barrier differences caused by the lack of IFNAR, such as alteration of neurons or the blood-brain barrier that affect viral trafficking, or, perhaps, a brain-specific IFNα/β response established by the first virus(es) to enter the brain contributes to the bottleneck observed in PVR mice. To determine whether the amount of virus entering the brain influences viral diversity, PVR and PVR-IFNAR^−/−^ mice were inoculated by the IC route with a low dose of the ten virus mixture, 2×10^3^ PFU, which corresponds to 200 PFU of each pool member. Using this low input dose, viral diversity was low in the brains of both PVR and PVR-IFNAR^−/−^ mice (13% and 24% of input viruses present, respectively) (Figure 2B). These results suggest that the bottleneck we observe is affected by the quantity of virus entering the brain.

The viral bottlenecks we observe are independent of selective advantages possessed by a particular marked virus. Based on a compilation of 479 hybridization signals, all ten viruses were approximately equally represented in a variety of tissues from over 25 mice, although virus 3 showed reduced representation, possibly indicating a slight growth defect (Figure 1D; Figure S3). However, statistical analysis revealed that none of the viruses, including virus 3, were significantly under- or over-represented in mouse tissues (p = 0.07 to p = 1, Student's *t* test). This apparent random sampling of population members was also observed in our previous study [24].

### Bottlenecks exist following oral inoculation of PVR-IFNAR^−/−^ mice

Unlike PVR mice, PVR-IFNAR^−/−^ mice are orally susceptible to poliovirus [22],[23]. Although the peripheral site-to-brain bottleneck was reduced in PVR-IFNAR^−/−^ mice (Figure 2A, 2B), we sought to determine whether bottlenecks exist following oral inoculation. Because the gut is a complex environment composed of many unique cell types and processes, barriers to viral spread may be encountered in PVR-IFNAR^−/−^ mice despite the hyper-susceptibility of these animals to poliovirus. We orally inoculated PVR-IFNAR^−/−^ mice with 2×10^7^ PFU of a mixture of the ten-member virus pool. Following oral inoculation, PVR-IFNAR^−/−^ mice developed encephalitis rather than paralysis observed in injected mice, and disease onset was delayed, with symptoms developing on days five through ten or later, in agreement with published data [23]. Feces were harvested daily from individual mice, and tissues were collected upon disease onset. Viruses isolated from stomach, small intestine, colon, feces, and blood were amplified for approximately three replication cycles in HeLa cells to increase detection, as the detection limit of the hybridization assay is ∼5,000 PFU (data not shown). *In vitro* amplification does not significantly affect diversity of virus extracted from tissues. For example, in the brain, where viral titers were high enough to perform the hybridization assay with or without amplification, viral diversity was equivalent in amplified and unamplified viral stocks (data not shown). Therefore, *in vitro* amplification allows detection without significantly altering the composition of the viral population.

**Figure 3:**
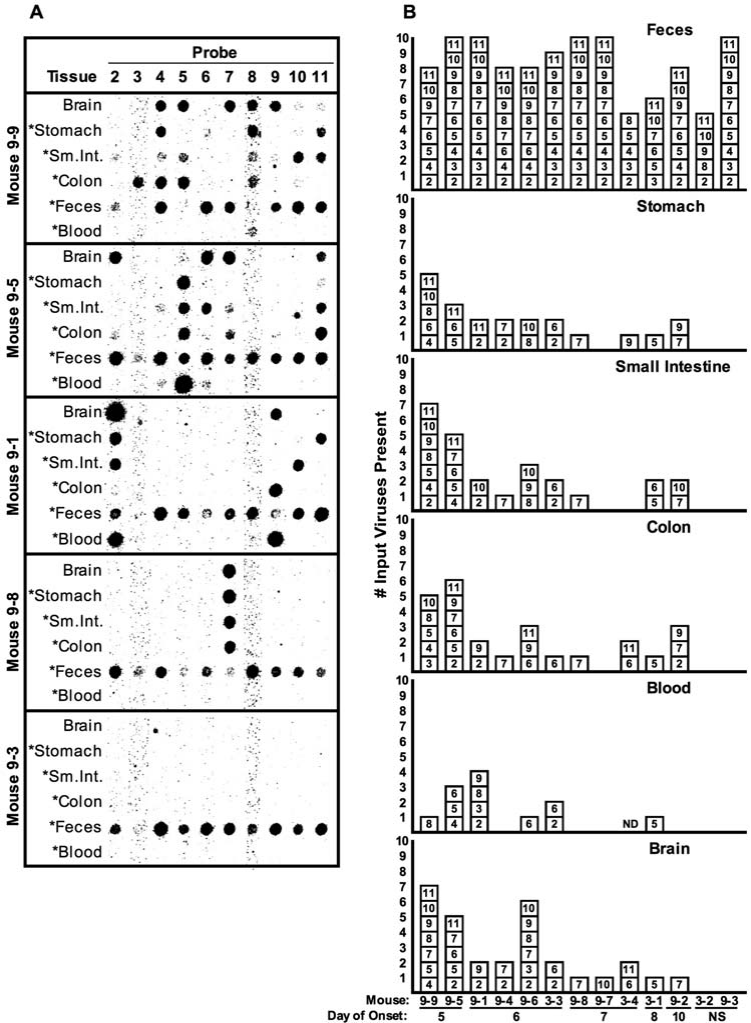
Viral diversity in orally inoculated PVR-IFNAR^−/−^ mice. Mice were orally inoculated with 2×10^7^ PFU of the ten-virus pool, and tissues were harvested upon disease onset. (A) Blot results for five representative orally inoculated PVR-IFNAR^−/−^ mice. Mouse 9-3 (bottom) did not develop disease during the ten-day post-inoculation time course. Sm.Int. = small intestine. Asterisks indicate samples that were the product of viral amplification in HeLa cells to improve detection (see text and Materials and Methods). (B) Compilation of input viruses found in each tissue at disease onset and found in feces collected on day one post-inoculation. Mice are arranged in order of earliest to latest disease onset (NS = no symptoms at harvest on day ten). Each box depicts a particular virus based on blot results. ND =  not determined.

Three major poliovirus bottlenecks were observed in orally inoculated PVR-IFNAR^−/−^ mice. First, a major bottleneck occurred between the inoculation site (mouth) and gut tissues (Figure 3). Gut tissues were harvested upon disease onset, and lumenal contents were removed. An average of approximately 20% of input viruses were present in the stomach, small intestine, and colon (Figure 3B). Notably, virus was detectable in the stomach late in infection upon disease onset, suggesting that non-input replicating virus was present. These results support the notion that poliovirus is resistant to stomach acid and digestive enzymes, although it is possible that viruses entered the bloodstream and re-seeded organs later in the disease course. Interestingly, viruses found in one GI tract tissue did not always correlate with those detected in other GI tract tissues within the same animal (e.g. mouse 9-1, Figure 3).

Second, a major bottleneck occurred between the mouth and blood (Figure 3). It is unclear how poliovirus enters the bloodstream, with evidence supporting upper GI and lower GI routes [34],[35],[36]. We found that less than 50% of mice had detectable virus in blood harvested at disease onset, with an average of 9% of input viruses present (Figure 3). Because it is likely that viremia occurred earlier in the disease course, we assessed viral population diversity in blood from a separate set of animals bled at several time points. Similar to the results obtained by sampling blood at disease onset, less than 60% of day three blood samples contained detectable virus, with an average of 17% of input viruses present (data not shown).

Third, a major bottleneck occurred between inoculation site and brain, with an average of 21% of input viruses detected in the brain, harvested upon disease onset (Figure 3). Surprisingly, viruses found in the brain did not always correlate with those detected in other tissues within the same animal.

Interestingly, the timing of disease onset and viral population diversity were associated, such that earlier disease onset correlated with higher diversity. Mice developing symptoms prior to day seven had 3.3-fold (p = 0.025) more input viruses in the brain than those developing symptoms after day seven, according to mean viral diversity comparison (Figure 3B). Higher diversity was also observed in blood and gut tissues of the early onset mice, with 9-fold higher diversity in blood (p = 0.042), and 2.1 to 3.3-fold higher diversity in gut tissues (stomach, small intestine, and colon; p<0.05).

### Passage through the GI tract is not difficult for poliovirus

With the finding that major bottlenecks occurred during viral trafficking from the mouth to other mouse tissues, it became important to determine whether transit through the gut environment is difficult for poliovirus populations. Interestingly, only a minimal bottleneck occurred between inoculation site (mouth) and feces (Figure 3A & 3B). For the population diversity assay, we analyzed fecal samples collected at 24 hours post-inoculation because relatively high viral titers were detected at this time. On average, more than 80% of input viruses were detected in feces (Figure 3B & 6A). Many of the mice (5/13) shed all ten input viruses in feces.

**Figure 4:**
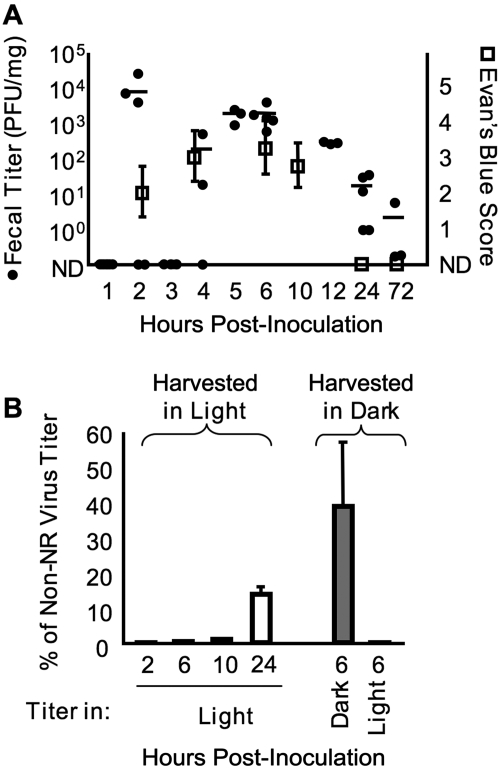
Kinetics of poliovirus shedding in feces. (A) Mice were orally inoculated with 2×10^7^ PFU of poliovirus or a solution of 8% Evan's Blue dye to trace transit through the GI tract. Fresh feces were harvested from individual mice at the indicated time points, and viral titers (filled circles) or Evan's Blue Score (open squares) were determined for 2–5 mice per time point. Titer averages are indicated by the horizontal lines. Evan's Blue Score was determined by assessing the level of blue dye in fecal samples (see Materials and Methods). ND, not detected (below the detection limit). (B) Neutral red (NR) light-sensitive poliovirus was used to measure input/non-replicated *vs.* replicated virus in feces. Mice were orally inoculated with 2×10^7^ PFU of NR-poliovirus in the dark, and feces were harvested in the dark (right side, gray bars) or in the light (left side, white bars) at the indicated time points. Fecal virus titers were determined in HeLa cells under light or dark conditions, and NR virus titers were divided by non-NR virus titers from the same time points (in panel A) to generate normalized titer values, expressed as “% of Non-NR Virus Titer”, an indication of the viral replication level prior to shedding in feces.

Because viral diversity was high in feces, we sought to determine whether the 24-hour fecal samples contained replicated virus, non-replicated/input virus, or both. First, we monitored viral transit time through the GI tract by measuring fecal titers at several time points, and transit time of a dye. Mice were orally inoculated with 2×10^7^ PFU of poliovirus, or Evan's Blue dye as a tracer. Fresh feces were harvested at regular intervals. Viral titers were determined by standard plaque assay using HeLa cells, and transit time of Evan's Blue was determined by scoring the relative dye intensity of fecal samples. As shown in Figure 4A, very high fecal titers were present at 2 hours post-inoculation for some animals. Since this time point is within the eclipse period of the viral replication cycle (see Figure S2), we presumed that virus shed at 2 hours post-inoculation was input/non-replicated virus. Viral titers remained relatively high from 5–12 hours post-inoculation, and then declined at later time points. This rise and decline of viral titers correlated well with the transit time of Evan's Blue dye through the mouse GI tract (Figure 4A).

Although the results from the fecal virus kinetics study suggested that virus shed at early time points is input/non-replicated virus, the presence of replicated virus could not be excluded; therefore, we monitored the transit of light-sensitive poliovirus to directly measure the amount of replicated *vs.* non-replicated virus present in feces. Poliovirus grown in the presence of neutral red (NR) is sensitive to inactivation by light exposure due to dye incorporation and concentration in virions [37],[38],[39]; hence, these viruses must be handled in the dark, using a red safety light. Upon replication in the absence of NR, viruses lose this light sensitivity. Therefore, the presence or absence of light-sensitive poliovirus in feces was utilized to monitor whether replication had occurred in the GI tract of orally inoculated mice. In the dark, mice were orally inoculated with 2×10^7^ PFU of light-sensitive NR-poliovirus, and feces were harvested in the light or in the dark. As a control, 6-hour feces harvested in the dark were subjected to titer analysis in light *vs.* dark conditions. The non-light exposed samples demonstrated high titers: viable virus titers were ∼40% of non-NR poliovirus titers harvested at the 6 hour time point in Figure 4A. We presume that these NR-virus titers were not 100% of the non-NR titers due to intrinsic variability in the animal experiments and/or subtle defects in NR-containing virions. Upon exposure to light, <0.1% of the non-NR poliovirus titer was obtained, indicating a very low level of light-insensitive viruses in the population (Figure 4B, right). Fecal samples exposed to light contained negligible viral titers until after 10 hours post-inoculation, suggesting that prior to 10 hours, feces contain input/non-replicated virus (Figure 4B, left). However, at 24 hours post-inoculation, feces contained light-insensitive/replicated virus, although only ∼14% of the non-NR poliovirus titer was obtained. Therefore, the 24-hour fecal samples used for our population diversity analysis contained a mixture of replicated and non-replicated/input virus.

### Effect of colonic mucosal damage and antacid administration on viral titers

**Figure 5:**
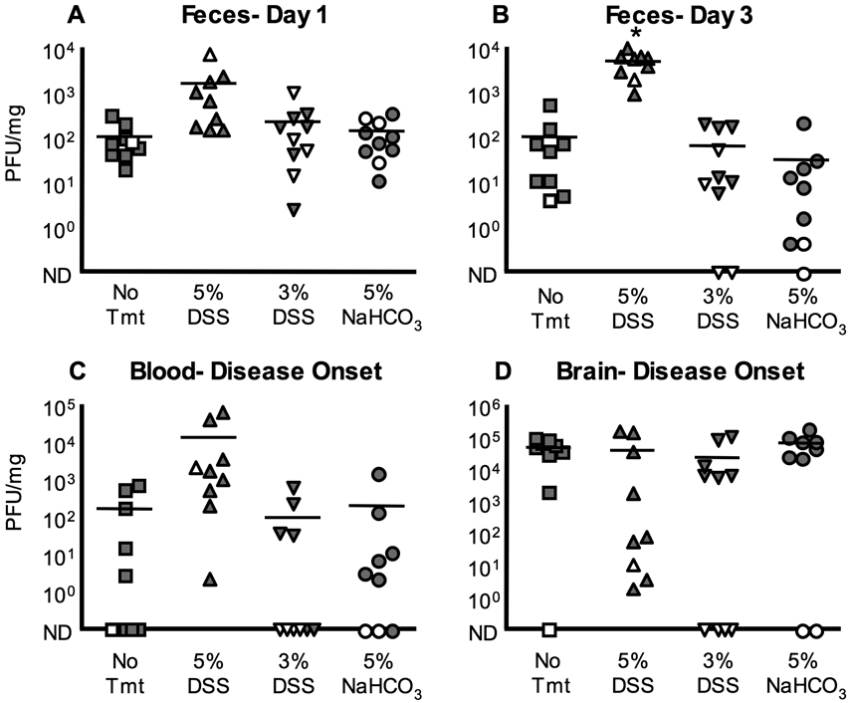
Poliovirus titers from orally inoculated untreated, DSS-treated, and antacid-treated PVR-IFNAR^−/−^ mice. Mice were left untreated or were pre-treated with 3% or 5% dextran sulfate sodium (DSS) in drinking water for 3 or 5 days, respectively. A group of mice were then infected with 2×10^7^ PFU of the ten-virus pool, in the presence or absence of an acid neutralizing agent, 5% NaHCO_3_. (A and B) Poliovirus titers, expressed as plaque-forming units (PFU) per mg, in feces harvested on day 1 (A) or day 3 (B) from untreated, 5% and 3% DSS-treated, and 5% NaHCO_3_-treated mice. Asterisk indicates statistically significant difference between untreated and 5% DSS treated mice (p<0.0002, Student's *t* test). (C) Poliovirus titers in blood collected at disease onset. (D) Poliovirus titers for brain collected at disease onset. Gray symbols = mice that developed disease; white symbols = mice that did not develop disease by day 10 post-inoculation. ND, not detected. The detection limit for the titer assay is ∼ 1 PFU/mg. Averages are indicated by horizontal lines.

We hypothesized that the colonic mucosal epithelium and/or stomach acidity may create barriers that contribute to viral bottlenecks. Therefore, we treated mice with agents that damage the colonic mucosa or neutralize stomach acid and determined the effects on poliovirus titer and diversity. Damage to the colonic mucosa was induced by treating mice with dextran sulfate sodium (DSS) in drinking water. DSS directly damages colonic epithelia resulting in ulceration, immune infiltration, and bloody feces [40],[41],[42],[43]. We measured viral titers in feces (Figure 5A, 5B), blood (Figure 5C), and brain (Figure 5D) following oral inoculation performed +/− DSS pre-treatment. High-dose (5%) DSS treatment increased 72-hour fecal titers 56-fold (p = 0.000163). Day one fecal titers were 16-fold higher in 5% DSS-treated mice compared to untreated mice. Blood titers for 5% DSS-treated mice were increased 66-fold, and virus was detected in the blood of all 5% DSS-treated mice (Figure 5C) compared to untreated mice, where less than 50% of animals had detectable virus in blood. Treatment with 3% DSS did not have an effect on viral titers suggesting that 3% DSS may not induce sufficient damage.

We next assessed the role of stomach acid in establishing the poliovirus bottleneck. The mouth-to-feces bottleneck is minor since the majority of the ten input viruses were detected in feces. However, Ohka and colleagues showed that sodium bicarbonate, an acid-neutralizing agent, increased poliovirus titers in a ligated stomach model following oral inoculation of PVR-IFNAR^−/−^ mice [23]. We orally inoculated PVR-IFNAR^−/−^ mice with a virus/5% sodium bicarbonate mixture. Our results revealed no titer differences between sodium bicarbonate-treated and untreated animals (Figure 5A–D).

### Colonic mucosal damage increases population diversity in GI tract and blood, but not brain

**Figure 6:**
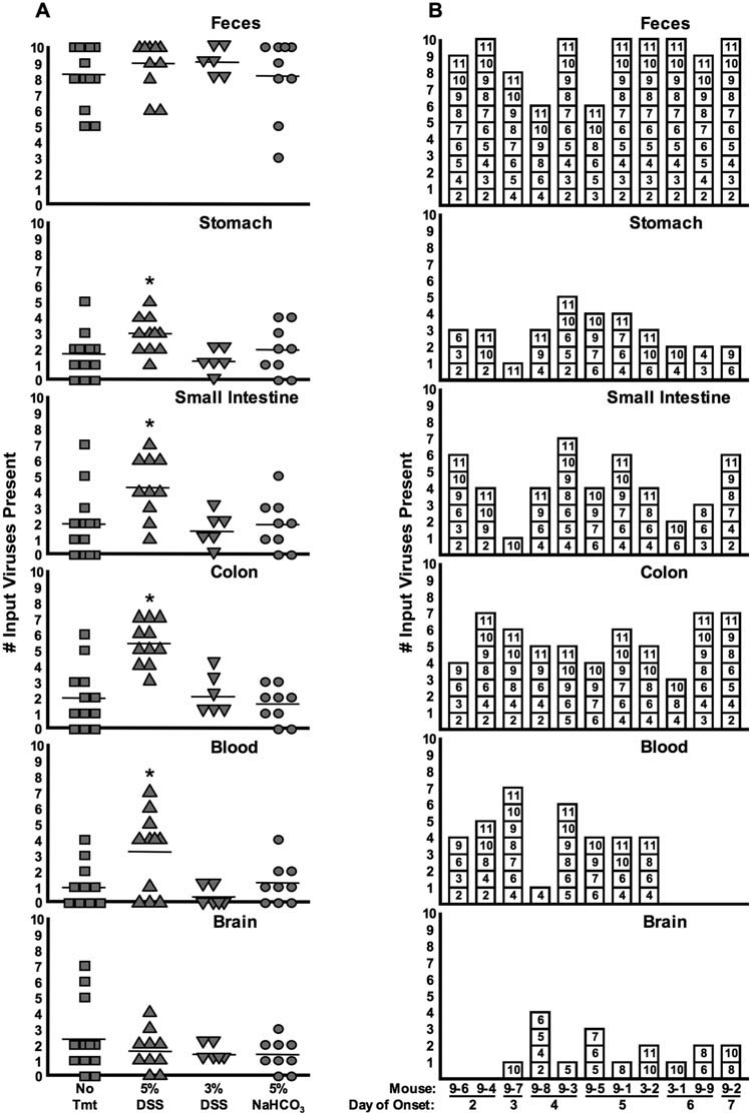
Viral diversity in tissues from orally inoculated untreated, DSS-treated, and antacid-treated PVR-IFNAR^−/−^ mice. Samples from the mice described in Figure 5 were subjected to the hybridization-based diversity assay. (A) Number of input viruses present, compiled from the hybridization-based assay, in different tissues and feces from untreated, 5% and 3% DSS-treated, and antacid (5% NaHCO_3_)-treated PVR-IFNAR^−/−^ mice. Averages are represented by horizontal lines. Asterisks indicate statistically significant differences between untreated and 5% DSS-treated mice (p<0.05, Student's *t* test; see text for exact p values). (B) Viral diversity in tissues of 5% DSS-treated mice. Shown here are individual viruses found in each tissue at disease onset and feces collected on day one post-inoculation. Each box depicts a particular virus detected following hybridization of probes, based on blot results. Mice are arranged in order of earliest to latest disease onset. It is likely that the first two mice, 9-6 and 9-4, succumbed to DSS treatment rather than poliovirus since no virus was detected in the brain of these animals.

Since 5% DSS-treated poliovirus-infected mice demonstrated increased viral titers, we reasoned that viral population diversity may be increased in these mice. Therefore, we performed the viral population diversity assay for samples from 5% DSS-treated, orally inoculated PVR-IFNAR^−/−^ mice. As expected, viral diversity in feces was high for all mice, regardless of treatment (Figure 6). Viral diversity in the stomach of 5% DSS-treated mice increased 1.8-fold (p = 0.0218), diversity in the small intestine increased 2.2-fold (p = 0.00865), and diversity in the colon increased 2.8-fold (p = 0.0000497) compared to untreated controls (Figure 6). Additionally, viral diversity in blood increased 3.5-fold (p = 0.0101). Interestingly, viral diversity in the brain was unaffected by DSS treatment (Figure 6A). Again, we found that viruses present in the brain do not necessarily correlate with those present in blood or gut tissues (Figure 6B). Viral diversity in tissues of mice treated with 3% DSS or sodium bicarbonate did not differ from untreated mice (Figure 6A).

### Comparison of viral titer vs. viral population diversity unmasks bottlenecks following oral inoculation

**Figure 7:**
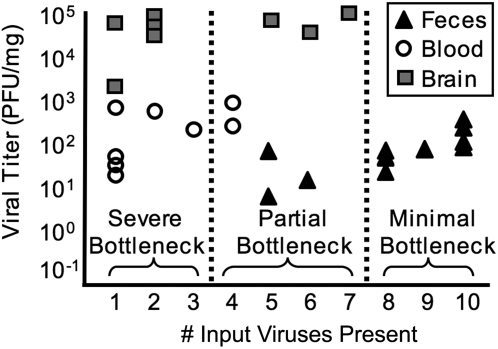
Bottlenecks revealed by comparing viral titer *vs.* diversity. Samples from untreated, orally inoculated PVR-IFNAR^−/−^ mice were compared by graphing viral titer (see Figure 5C–F) *vs.* the number of input viruses present (see Figure 6A). Samples with 1–3 input viruses present experienced a severe bottleneck, samples with 4–7 input viruses present experienced a partial bottleneck, and samples with 8–10 input viruses present experienced a minimal bottleneck.

Initially, one might assume that viral titer and viral diversity are linked, with high titer sites containing high population diversity, and vice versa. However, this is not the case, especially when bottlenecks are present [25],[44]. Figure 7 compares viral titer *vs.* diversity for feces, blood, and brain viruses from untreated mice orally inoculated with the ten-virus mixture. Fecal samples contained low to moderate titers of ∼5–300 PFU/mg, but contained moderate to high population diversity. Titer and diversity may be linked before a major bottleneck is encountered, as in feces, in which higher titers correlate with higher diversity. These results confirmed that the bottleneck between mouth and feces is minor. Brain samples had the highest titers (∼2,000–100,000 PFU/mg), but contained low diversity, which is characteristic of a major bottleneck. We propose that entry into the brain is difficult, but once in the brain, founder viruses undergo robust replication. Blood samples had low to moderate titers (∼1–700 PFU/mg) and low diversity. Therefore, our data confirm that titer and diversity are not linked following bottlenecks.

## Discussion

We have developed a new diversity assay that has allowed us to uncover barriers to viral trafficking that would be missed by standard viral titer assays. Using our hybridization-based assay, we demonstrated bottleneck barriers by monitoring marked polioviruses.

We confirmed a previously observed bottleneck between peripheral injection sites and brain (Figure 2). As before, random sampling was revealed, in which no pool member had an apparent selective advantage over the others (Figure 1D; Figure S3). The previous assay employed four viruses, and one to three were found in the brain (average ∼50%) following IM injection [24]. Here we found that, on average, ∼20% of our ten marked viruses reached the brain, suggesting that this bottleneck was more severe than that previously observed. One possible explanation is that the previous study was performed using ICR-PVR mice [18], while this study was performed using C57/BL6-PVR mice [45]. Additionally, the observed increase in bottleneck severity could be a result of our increased artificial quasispecies sample size.

Because interferons (IFN) play an important role in controlling viral infections, prior to this study, we proposed that the IFNα/β response may contribute to viral bottlenecks. In PVR-IFNAR^−/−^ mice, the bottleneck following IM or IP injection was largely absent with an average of 70% or 80% of input viruses detected in the brain, respectively. In fact, direct injection of a large inoculum (2×10^7^ PFU) of the virus pool into the brains of PVR mice resulted in an average of 76% of input viruses detected in the brain, confirming the absence of a major bottleneck in peripherally-injected PVR-IFNAR^−/−^ mice. We propose several possible reasons for the diminished bottleneck in peripherally-injected PVR-IFNAR^−/−^ mice: 1) The first viruses to enter the brain in PVR mice established an anti-viral state which limited the spread of later viruses, resulting in a bottleneck effect. The lack of IFNα/β response in PVR-IFNAR^−/−^ mice, therefore, facilitated higher brain diversity. 2) Increased peripheral titers in hyper-susceptible PVR-IFNAR^−/−^ mice may have essentially increased the poliovirus dose. This effect could be unique to PVR-IFNAR^−/−^ mice since our previous work determined it was very difficult to overcome the bottleneck by increased dose in PVR mice [24]. 3) Physical barriers in PVR-IFNAR^−/−^ mice may have been altered due to lack of the type I IFN environment. Perhaps lack of IFNAR created differences in neurons or the blood-brain barrier that may have contributed to higher viral brain diversity in PVR-IFNAR^−/−^ mice. Importantly, data from our oral inoculation studies demonstrated a bottleneck exists between mouth and brain in PVR-IFNAR^−/−^ mice (Figure 3). Therefore, the lack of the IFNα/β response in the brain was not the sole cause for the diminished bottleneck in peripherally-injected PVR-IFNAR^−/−^ brains. Additionally, PVR and PVR-IFNAR^−/−^ mice injected by the IC route with a low dose of the virus pool (2×10^3^ PFU) demonstrated comparable low levels of viral diversity in the brain (13% and 24% of input viruses, respectively). These results suggest that viral diversity in the brain is governed by the amount of virus that enters the brain, and that elevated peripheral titers in injected PVR-IFNAR^−/−^ mice contribute to the elevated viral diversity in the brains of these animals.

Following oral inoculation of PVR-IFNAR^−/−^ mice, poliovirus moves through the GI tract without much difficulty. Relatively high amounts of virus were shed in feces, including input/non-replicated viruses and replicated viruses, depending on the sampling time (Figure 4). Population diversity in feces was relatively high with an overall average of 81% of input viruses present (Figures 3 and 6A), suggesting only a minor bottleneck was encountered during GI lumenal passage. Although we consider this bottleneck minor, it could actually represent the successful passage of just 0.025% (5×10^3^ PFU) of the input virus, which would still allow detection of all pool members in our system. Regardless, this mouth-to-feces bottleneck was minor in comparison to other bottlenecks we observed.

Our experiments identified three major bottlenecks following oral inoculation of PVR-IFNAR^−/−^ mice: mouth-to-gut tissues, mouth-to-blood, and mouth-to-brain. First, a major bottleneck existed between the mouth and gut tissues. Of the ten viruses, an average of 16% of input viruses were present in the stomach, and 19% of input viruses were present in the small intestine and the colon (Figures 3 and 6). We presume that virus must be replicating in these tissues to be detected late in infection when the tissues were harvested (day 5–10). However, gut tissues could have been re-seeded by virus in the blood.

We identified a second bottleneck between mouth and blood. Blood titers were moderate, but diversity was very low (avg. = 9% of input viruses) (Figures 3, 6, and 7). We are uncertain how the virus is traveling from the inoculation site into the blood, but possibilities include drainage from lymph, mucosal passage to the blood, and entrance into the bloodstream at sites of mucosal micro-damage. Viruses may have entered the bloodstream early in infection [34],[46],[47].

Third, a prominent bottleneck existed between the mouth and the brain. Viral trafficking between the mouth and brain could have occurred through blood or neural routes. Historically, poliovirus invasion of the brain has been presumed to occur through the blood route because neutralizing antibodies are protective and IV-injected radiolabeled virions readily enter the murine brain [21],[48],[49]. However, viral trafficking in neurons may also occur and contribute to pathogenesis [10],[19],[20],[50].

Surprisingly, 93% of viruses found in the brain were present in gut tissues of a given mouse, but only 35% were detected in blood (Figure 3B). This suggests that a gut tissue-to-brain pathway was involved in viral spread. Virus may have entered the blood from gut tissues and trafficked to the brain, or virus may have infected neurons associated with the GI tract and reached the brain by retrograde transport. Trafficking via neurons has been demonstrated by sciatic nerve transection experiments following poliovirus infection [19],[20]. Although our data did not definitively discriminate between blood and neural routes, our data did show that absolute match of blood and brain viruses was rare. In some instances, there was no overlap between viruses present in the brain and blood (Figure 3B, 9-6; Figure 5, 9-1). These results indicated that virus may enter the brain by a non-hematogenous route, such as neurons, that low-abundance viruses in blood seeded the brain, or that virus found in the blood at disease onset differed from the virus in the blood at earlier time-points. Interestingly, the blood/brain virus mismatch was confirmed in an experiment where blood was collected at day three post-inoculation and upon disease onset, and then blood diversity was compared with brain diversity. This experiment revealed that only 44% of viruses found in the brain are present in blood at day three post-infection, suggesting that not all viruses may spread to the brain via a blood route (data not shown). Aside from possible GI neuronal trafficking, it is likely that virus moves into and out of blood throughout infection by seeding other tissues with subsequent re-seeding of the blood. In humans, it is thought that a primary asymptomatic viremia may seed tissues, with a subsequent secondary viremia contributing to minor or major illness, which can lead to CNS invasion and paralytic poliomyelitis [34],[35],[36].

Our results suggested that disease onset and viral diversity are linked. Earlier disease onset correlated with greater viral diversity in the gut tissues, blood, and brain. Mice that developed symptoms before day seven had 3.3-fold (p = 0.025) higher brain diversity than those that developed symptoms later (Figure 3B). These early onset mice also had higher blood diversity (9-fold, p = 0.042) and gut diversity (2.1–3.3 fold, stomach: p<0.05). Greater diversity upon earlier onset was not simply due to a tissue sampling time bias, because a separate study demonstrated very low population diversity in tissues harvested on days one and three post-inoculation (Figure S4). There are several possible explanations for the correlation of disease onset and viral diversity. First, since high population diversity and virulence are linked [4],[5], higher viral diversity may contribute to faster disease progression. Second, some component of host immunity may have developed later in infection, which limited viral replication, and ultimately, viral diversity.

Interestingly, two of the three major bottlenecks could be overcome by pre-treating the mice with a colonic epithelial-damaging agent, DSS. The first (mouth-to-gut tissues) and second bottlenecks (mouth-to-blood) were affected by DSS treatment: gut tissue and blood diversity increased ∼2–3-fold and 3.5-fold, respectively. Additionally, blood titers increased 66-fold in the presence of colonic damage. Importantly, the mouth-to-brain bottleneck was unchanged in DSS-treated mice compared to untreated mice. These results suggest that either virus trafficked to the brain via a non-blood route, which was unaffected by DSS treatment, or virus trafficked to the brain via a blood route, but spread to the brain was limited by another barrier, such as the blood-brain barrier.

Viral titer and diversity were not linked after a bottleneck was encountered (Figure 7). By monitoring diversity, we uncovered limitations on viral trafficking that would be missed by viral titer analysis. For example, blood and fecal titers were similar; therefore, one might conclude that transit from the gut to blood was not difficult. Our assay allowed us to conclude that a major bottleneck exists since blood diversity was low. We presume that virus was replicating in blood and/or other tissues that seed blood, thus increasing the blood titer post-bottleneck encounter, resulting in founder effects.

We found that viral population trafficking was a very dynamic, stochastic process. Using virus 2 as an example, a given virus might be present in all tissues (Figure 3, 9-1), in colon and feces only (Figure 3, 9-2), in brain and feces only (Figure 3, 9-6), or in other differing combinations. Similar random trafficking patterns have been observed in several microbial systems, including animal and plant viruses, bacteria, and fungi [25],[26],[27],[28],[29],[30],[31],[32]. For highly mutable RNA viruses, host barriers likely play an important role in shaping viral populations and determining virulence [51].

The random distribution of viral populations makes predicting VAPP (vaccine-associated paralytic poliomyelitis) impossible, because a viral isolate from the CNS of one person may not invade the CNS of another due to bottleneck effects and stochastic trafficking. Notably, in human VAPP patients, fecal virus does not always correlate with virus found in the CNS [15]. Perhaps physical barrier disruption and/or a defective innate immune response increased susceptibility to inadvertent poliovirus CNS invasion in individuals afflicted with paralytic poliomyelitis. We have shown that this artificial quasispecies system mimics the stochastic poliovirus trafficking observed in humans, and can be used to understand RNA virus population dynamics in an infected host.

## Materials and Methods

### Plasmid Construction

The ten viral plasmids (2 through 11) were made using silent site-directed mutagenesis of the Mahoney serotype 1 viral cDNA clone beginning with nucleotide 2425 and ending at 2443 (Figure 1A) [52]. Two unique silent restriction sites were added, Bgl II at nucleotide 5601 and Mlu I at nucleotide 7550, in order to facilitate cloning. Each PCR-generated region was confirmed by sequencing (Sequencing Core, UT Southwestern Medical Center, Dallas, TX).

### Viruses and Cell Culture Infections

All poliovirus work was done in WHO-approved elevated BSL2/poliovirus conditions. Cell culture infections and propagation of virus was performed from a single poliovirus plaque using HeLa cells grown in Dulbecco's modified Eagle's medium with 10% calf serum as previously described [53]. For the viral serial passage experiment (Figure 1D), the ten viruses were combined at equivalent amounts and single-cycle infections beginning with a MOI of 0.1, were performed as described [4]. Virus stocks were titered using plaque assays in HeLa cells as previously described [53]. A neutral red (NR)-poliovirus stock was prepared by infecting HeLa cells with wild-type poliovirus in the presence of 10 ug/ml neutral red (Sigma) in the dark, using a red safety light [37],[38],[39]. NR-poliovirus stocks were light inactivated by exposure to a fluorescent light bulb at a distance of 3 inches for 10 minutes. The ratio of light-insensitive to light-sensitive PFU in the NR-poliovirus stock was 1 to 1.27×10^6^.

### Mice, Treatments, and Infections

All animal work was performed according to protocols approved by the UT Southwestern Medical Center IACUC. C57/BL6 PVR-Tg21 (PVR) mice and C57/BL6 PVR-IFNAR^−/−^ (PVR-IFNAR^−/−^) mice were obtained from S. Koike (Tokyo, Japan), and maintained in specific pathogen free conditions [22]. Intramuscular (50 µl volume) and intracerebral (15 µl volume) injections were done as previously described [4] using 2×10^7^ PFU total (2×10^6^ PFU of each of the 10 viruses), or 2×10^3^ PFU total for low-dose IC injections. For intraperitoneal injections, 1×10^8^ PFU total of the 10 viruses were injected in a volume of 50 µl. It should be noted that inocula for all experiments in this study were based on viral titers obtained using HeLa cells. We have shown previously that poliovirus titers in PVR-derived mouse embryo fibroblasts (PVR-MEFs) are approximately 300-fold lower than those obtained in HeLa cells [4]. Therefore, in terms of poliovirus titers in mouse cells, mice were actually inoculated with 6.67×10^4^ PFU for the “2×10^7^ PFU” inoculations. Oral inoculations were performed by dispensing 15 µl of virus, by pipette tip, in the mouth. Each mouse was euthanized at first signs of disease, which included encephalitis, ruffled fur, lethargy, and paralysis. In our experience, once symptoms develop, the mice die within a day. For DSS treatments, mice were pre-treated with DSS (molecular weight 36,000–50,000; MP Biomedicals LLC, Solon, OH) in their drinking water prior to oral inoculation [40]. Mice receiving 3% DSS were pre-treated for three days, and mice receiving 5% DSS were pre-treated for five days. Once infections were performed, the mice were provided with regular drinking water for the course of the experiment. Sodium bicarbonate was added to virus to make 5% mixtures immediately prior to oral infections [23]. Mice were housed in individual cages and feces were collected at 24-hour intervals with subsequent bedding changes. A combination of moist, freshly acquired feces and dry feces were combined to generate the fecal samples for the population diversity assay. For kinetics of viral shedding experiments (Figure 4), fresh feces were harvested from each mouse. For Evan's Blue dye transit experiments (Figure 4A), feces were weighed, resuspended in 6 volumes of PBS, freeze-thawed three times, and samples were centrifuged at 13,000 rpm for 1 minute. “Evan's Blue Score” was determined by assessing the level of blue color in the feces: slightly blue = 1, light blue = 2, moderate blue = 3, dark blue = 4, intense blue = 5. Upon euthanasia, blood, stomach, small intestine, colon, and brain were harvested and stored at −80°C prior to use. During tissue harvests, lumenal contents were removed from gut tissues.

### Sample Processing and Hybridization-based Viral Diversity Assay

Tissues (brain, stomach, small intestine, colon) were homogenized under liquid nitrogen using a mortar and pestle. For brain RNA extractions, 1 ml of TRIZOL (Invitrogen, Carlsbad, CA) was added to approximately 300mg of tissue, and extractions and RT-PCR were performed as previously described [4]. BN2 antisense primer 5′ ATGCTTTCAAGCATCTGACCTAACC 3′ and NdeI sense primer 5′ AAACTGTTGGTGTCATATGCGCCTCCTGGAG 3′ were used for RT-PCR and PCR. To amplify virus from tissues, homogenized tissues were weighed and resuspended in 3 volumes of PBS+ (1× PBS supplemented with 100 µg/ml MgCl_2_ and CaCl_2_), and freeze-thawed 3 times. Feces were weighed, resuspended in PBS, and freeze-thawed three times. Each tissue slurry was dounce homogenized and centrifuged at 13,000 rpm for 1 minute, and supernatants were kept as virus stocks. To limit microbial contamination, virus from gut samples (stomach, small intestine, colon, and feces) were chloroform extracted by adding 1/10 volume of chloroform, centrifuged at 13,000 rpm for 2 minutes, and the supernatant was kept as the virus stock. Virus was amplified for 2–3 rounds of replication (12–16 hours) at 37°C in HeLa cells and the cells were harvested, resuspended in 50–100 µl of PBS+, freeze-thawed, and kept as amplified virus stock. Half of the amplified virus stock was added to 1ml of TRIZOL for RNA extractions and RT-PCR. PCR was performed in quadruplicate and products were combined before they were run on an agarose gel and quantitated by standards of known concentrations. These concentrations were used to normalize the amount of PCR product blotted to 50–100 ng of PCR product for each sample. DNA was blotted onto Hybond N+ membranes (GE Healthcare, Buckinghamshire, UK) using a 96-well vacuum manifold, and membranes were pre-hybridized and hybridized following standard procedures [54]. Optimal hybridization annealing temperature was empirically determined to be 59°C (data not shown). Probes were made by kinase treatment of specific primers (see Figure S1) with [γ-^32^P] ATP and excess nucleotides were removed with the Qiagen Nucleotide Removal kit (Qiagen, Valencia, CA) [4]. Membranes were exposed to PhosphorImager screens and scanned by Stormscan. Scanned blots were normalized by comparison of equivalently loaded products of perfectly matched PCR product to probe or mismatched PCR products to probe. Blot image intensities were adjusted such that any apparent mismatch dot was no longer visible, thus eliminating the minimal level of cross-reactivity of the probes with non-matched PCR products (Figure 1E).

### Accession Number

The Genbank (http://www.ncbi.nlm.nih.gov/entrez) accession number for serotype 1 poliovirus (Mahoney strain) is NC002058.

## Supporting Information

Figure S1 Oligonucleotide probe sequences for detection of individual virus pool members.
(0.40 MB PDF)

Figure S2 Single-cycle growth curves.
(0.30 MB PDF)

Figure S3 Distribution of pool members in mouse tissues.
(0.25 MB PDF)

Figure S4 Viral diversity in tissues harvested at early time points.
(2.58 MB TIF)
