# Supplementary material for: Multiple Host Barriers Restrict Poliovirus Trafficking in Mice
Source: PLoS Pathog. 2008 Jun 6;4(6):e1000082. doi: 10.1371/journal.ppat.1000082 (PMC2390757; doi:10.1371/journal.ppat.1000082)
Supplement: Figure S1 — Oligonucleotide probe sequences for detection of individual virus pool members. (0.40 MB PDF) [file ppat.1000082.s001.pdf]

Primer		Sequence	
CEBN2anti	5'	TCAGTGTCAGGCTCTTGCGAG	3'
CEBN3anti	5'	TCAGCGTTAGACTACTGCGAG	3'
CEBN4anti	5'	TCAGTGTGCGTTTGCTGAGAG	3'
CEBN5anti	5'	TCAGTGTTCGGTTACTACGGG	3'
CEBN6anti	5'	TTAGCGTACGCTTGCTCAGGG	3'
CEBN7anti	5'	TCAGCGTACGGCTATTACGTG	3'
CEBN8anti	5'	TTAGCGTCAGGTTGTTGAGAG	3'
CEBN9anti	5'	TCTCGGTGCGTTTACTTCGTG	3'
CEBN10anti	5'	TCAGTGTGAGATTATTGAGGG	3'
CEBN11anti	5'	TTTCTGTTCGCTTGTTGCGAG	3'

Supplemental Figure S1. Oligonucleotide probe sequences for detection of individual virus pool members.
