# Supplementary material for: Multiple Host Barriers Restrict Poliovirus Trafficking in Mice
Source: PLoS Pathog. 2008 Jun 6;4(6):e1000082. doi: 10.1371/journal.ppat.1000082 (PMC2390757; doi:10.1371/journal.ppat.1000082)
Supplement: Figure S2 — Single-cycle growth curves. (0.30 MB PDF) [file ppat.1000082.s002.pdf]

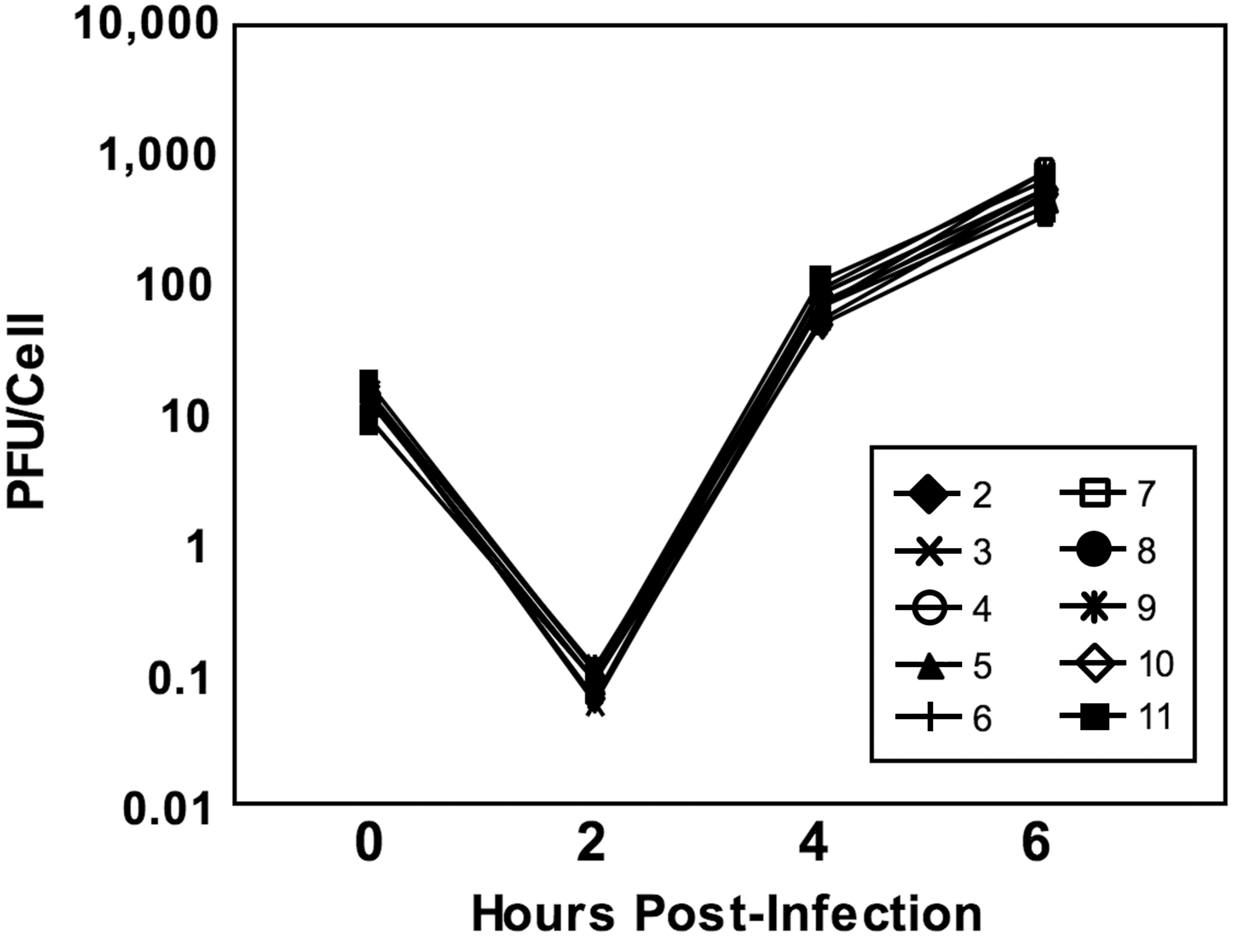

Supplemental Figure S2. Single-cycle growth curves. HeLa cells were infected using an MOI of 10 PFU/cell, and cell-associated virus was harvested at the indicated time-points and titered on fresh HeLa cells.
