# Supplementary material for: Multiple Host Barriers Restrict Poliovirus Trafficking in Mice
Source: PLoS Pathog. 2008 Jun 6;4(6):e1000082. doi: 10.1371/journal.ppat.1000082 (PMC2390757; doi:10.1371/journal.ppat.1000082)
Supplement: Figure S3 — Distribution of pool members in mouse tissues. (0.25 MB PDF) [file ppat.1000082.s003.pdf]

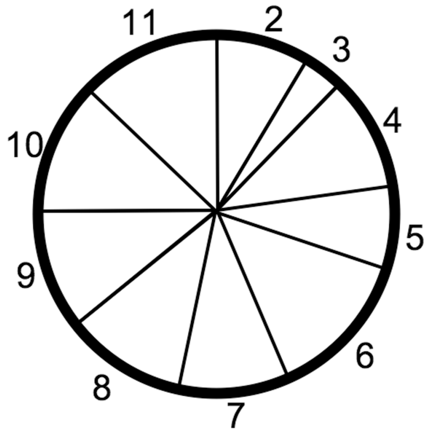

Supplemental Figure S3. Distribution of pool members in mouse tissues. Data derived from 479 hybridization dots representing over 25 mice were compiled into a pie graph. The numbers around the outside indicate the virus # (see Fig. 1).
