# Supplementary figures and images for: Multiple Host Barriers Restrict Poliovirus Trafficking in Mice
Source: PLoS Pathog. 2008 Jun 6;4(6):e1000082. doi: 10.1371/journal.ppat.1000082 (PMC2390757; doi:10.1371/journal.ppat.1000082)

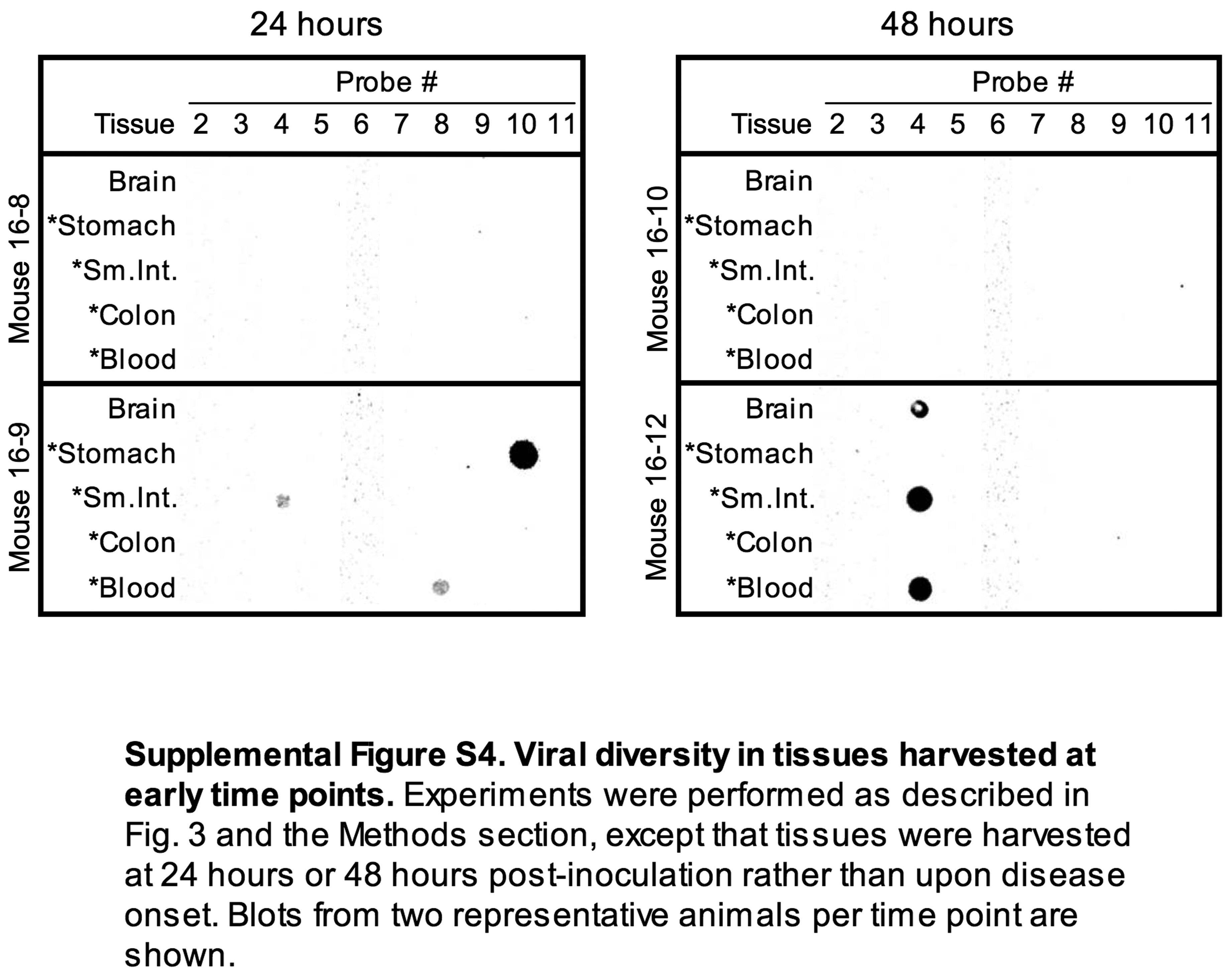

Supplement: Figure S4 — Viral diversity in tissues harvested at early time points. (2.58 MB TIF) [file ppat.1000082.s004.tif]
